# Supplementary material for: Hot spot prediction in protein-protein interactions by an ensemble system
Source: BMC Syst Biol. 2018 Dec 31;12(Suppl 9):132. doi: 10.1186/s12918-018-0665-8 (PMC6311905; doi:10.1186/s12918-018-0665-8)
Supplement: Supplementary file 2 — All relevant MATLAB code. A simple Matlab implement of our predictor. (ZIP 19,742 kb) [file 12918_2018_665_MOESM2_ESM.zip › Additional file 2/Read ASA/ASA website/dbMPIKT/3K2MD.html]

 NetSurfP - Protein Surface Accessibility and Secondary Structure Predictions

  

|  |  |  |
| --- | --- | --- |
|  |  | NetSurfP - Protein Surface Accessibility and Secondary Structure Predictions  Technical University of Denmark |

  


---

```
# For publication of results, please cite:
# A generic method for assignment of reliability scores applied to solvent accessibility predictions.
# Bent Petersen, Thomas Nordahl Petersen, Pernille Andersen, Morten Nielsen and Claus Lundegaard
# BMC Structural Biology 2009, 9:51 doi:10.1186/1472-6807-9-51
#
# Column 1: Class assignment - B for buried or E for Exposed - Threshold: 25% exposure, but not based on RSA
# Column 2: Amino acid
# Column 3: Sequence name
# Column 4: Amino acid number
# Column 5: Relative Surface Accessibility - RSA
# Column 6: Absolute Surface Accessibility
# Column 7: Z-fit score for RSA prediction
# Column 8: Probability for Alpha-Helix
# Column 9: Probability for Beta-strand
# Column 10: Probability for Coil
E G  3K2MD                  1    0.901  70.909  -1.435   0.003   0.003   0.994
E S  3K2MD                  2    0.635  74.457  -0.645   0.018   0.088   0.893
E S  3K2MD                  3    0.601  70.425  -1.048   0.018   0.088   0.893
E V  3K2MD                  4    0.419  64.370  -0.408   0.018   0.047   0.935
E S  3K2MD                  5    0.344  40.293  -1.067   0.018   0.047   0.935
E S  3K2MD                  6    0.647  75.864  -0.408   0.018   0.047   0.935
E V  3K2MD                  7    0.368  56.562   0.221   0.018   0.019   0.964
B P  3K2MD                  8    0.192  27.216  -1.434   0.018   0.019   0.964
E T  3K2MD                  9    0.497  68.906  -0.081   0.018   0.047   0.935
E K  3K2MD                 10    0.503 103.426   0.194   0.004   0.138   0.858
B L  3K2MD                 11    0.068  12.524   0.418   0.004   0.514   0.481
E E  3K2MD                 12    0.464  81.078   0.886   0.001   0.900   0.099
B V  3K2MD                 13    0.181  27.866  -0.546   0.001   0.959   0.040
E V  3K2MD                 14    0.389  59.789  -0.009   0.001   0.900   0.099
E A  3K2MD                 15    0.517  56.940  -1.134   0.003   0.718   0.279
E A  3K2MD                 16    0.389  42.835  -0.283   0.004   0.514   0.481
E T  3K2MD                 17    0.395  54.759  -0.326   0.004   0.138   0.858
E P  3K2MD                 18    0.525  74.441  -0.176   0.005   0.045   0.951
E T  3K2MD                 19    0.452  62.665  -0.138   0.005   0.262   0.733
B S  3K2MD                 20    0.226  26.452   0.043   0.002   0.816   0.182
B L  3K2MD                 21    0.040   7.269  -0.006   0.001   0.900   0.099
E L  3K2MD                 22    0.337  61.686   1.634   0.001   0.959   0.040
B I  3K2MD                 23    0.046   8.547   0.604   0.001   0.959   0.040
B S  3K2MD                 24    0.219  25.690   0.130   0.001   0.959   0.040
B W  3K2MD                 25    0.057  13.781  -0.111   0.002   0.816   0.182
E D  3K2MD                 26    0.491  70.695   1.195   0.004   0.616   0.381
E A  3K2MD                 27    0.311  34.217   0.280   0.005   0.262   0.733
B P  3K2MD                 28    0.296  41.988  -0.385   0.005   0.045   0.951
E M  3K2MD                 29    0.716 143.352  -1.030   0.005   0.015   0.979
E S  3K2MD                 30    0.509  59.643  -1.093   0.005   0.015   0.979
E S  3K2MD                 31    0.678  79.450  -1.848   0.005   0.015   0.979
E S  3K2MD                 32    0.456  53.490  -1.893   0.005   0.045   0.951
E S  3K2MD                 33    0.465  54.545   0.433   0.004   0.197   0.799
B V  3K2MD                 34    0.101  15.508   0.024   0.004   0.616   0.381
E Y  3K2MD                 35    0.362  77.338   0.517   0.002   0.816   0.182
E Y  3K2MD                 36    0.288  61.546   0.839   0.001   0.900   0.099
B Y  3K2MD                 37    0.048  10.300   0.606   0.001   0.959   0.040
B R  3K2MD                 38    0.224  51.388   1.694   0.001   0.959   0.040
B I  3K2MD                 39    0.028   5.217   0.589   0.001   0.959   0.040
B T  3K2MD                 40    0.194  26.908   1.216   0.001   0.959   0.040
B Y  3K2MD                 41    0.093  19.831   1.027   0.001   0.959   0.040
E G  3K2MD                 42    0.287  22.548   0.868   0.001   0.959   0.040
E E  3K2MD                 43    0.356  62.123  -0.615   0.003   0.718   0.279
E T  3K2MD                 44    0.380  52.748  -1.006   0.004   0.138   0.858
E G  3K2MD                 45    0.561  44.166  -1.360   0.005   0.015   0.979
E G  3K2MD                 46    0.647  50.935  -2.183   0.005   0.015   0.979
E N  3K2MD                 47    0.691 101.148  -2.021   0.005   0.015   0.979
E S  3K2MD                 48    0.539  63.147  -1.270   0.005   0.045   0.951
E P  3K2MD                 49    0.363  51.439   0.128   0.004   0.197   0.799
E V  3K2MD                 50    0.433  66.583  -0.386   0.004   0.616   0.381
E Q  3K2MD                 51    0.374  66.796   0.703   0.002   0.816   0.182
E E  3K2MD                 52    0.455  79.471   0.448   0.001   0.959   0.040
B F  3K2MD                 53    0.141  28.339   0.199   0.001   0.959   0.040
E T  3K2MD                 54    0.498  69.017   0.240   0.001   0.959   0.040
B V  3K2MD                 55    0.099  15.201  -0.528   0.002   0.816   0.182
E P  3K2MD                 56    0.515  73.036   0.052   0.005   0.262   0.733
E Y  3K2MD                 57    0.448  95.673  -1.172   0.005   0.015   0.979
E S  3K2MD                 58    0.668  78.266  -0.605   0.018   0.047   0.935
E S  3K2MD                 59    0.472  55.377   0.660   0.004   0.197   0.799
B S  3K2MD                 60    0.246  28.796   0.124   0.021   0.451   0.528
E T  3K2MD                 61    0.352  48.892   0.819   0.018   0.846   0.136
B A  3K2MD                 62    0.118  12.960   0.981   0.006   0.962   0.032
E T  3K2MD                 63    0.354  49.058   1.765   0.011   0.918   0.071
B I  3K2MD                 64    0.064  11.840   0.692   0.018   0.846   0.136
E S  3K2MD                 65    0.543  63.616   1.225   0.022   0.359   0.619
E G  3K2MD                 66    0.554  43.592   0.369   0.019   0.141   0.840
B L  3K2MD                 67    0.088  16.186   0.511   0.018   0.088   0.893
E S  3K2MD                 68    0.478  56.022   0.830   0.005   0.045   0.951
E P  3K2MD                 69    0.564  80.088   0.042   0.005   0.045   0.951
E G  3K2MD                 70    0.578  45.457  -0.991   0.005   0.015   0.979
B V  3K2MD                 71    0.222  34.137   0.031   0.004   0.138   0.858
E D  3K2MD                 72    0.482  69.499   1.334   0.003   0.718   0.279
B Y  3K2MD                 73    0.073  15.515   0.906   0.001   0.959   0.040
E T  3K2MD                 74    0.293  40.653   2.096   0.001   0.959   0.040
B I  3K2MD                 75    0.027   5.032   0.887   0.001   0.959   0.040
B T  3K2MD                 76    0.133  18.433   1.650   0.001   0.959   0.040
B V  3K2MD                 77    0.016   2.521   1.595   0.001   0.959   0.040
B Y  3K2MD                 78    0.156  33.316   1.652   0.001   0.959   0.040
B A  3K2MD                 79    0.016   1.807   0.813   0.001   0.959   0.040
B W  3K2MD                 80    0.129  30.952   0.279   0.001   0.959   0.040
B G  3K2MD                 81    0.207  16.315  -0.324   0.002   0.816   0.182
E E  3K2MD                 82    0.522  91.141  -0.573   0.005   0.262   0.733
E D  3K2MD                 83    0.396  57.006  -1.550   0.018   0.047   0.935
E S  3K2MD                 84    0.493  57.815  -2.202   0.018   0.047   0.935
E A  3K2MD                 85    0.535  58.957  -2.338   0.018   0.047   0.935
E G  3K2MD                 86    0.417  32.786  -2.011   0.018   0.047   0.935
E Y  3K2MD                 87    0.569 121.595  -2.195   0.018   0.047   0.935
E M  3K2MD                 88    0.523 104.612  -1.861   0.018   0.088   0.893
E F  3K2MD                 89    0.389  77.992  -2.223   0.019   0.141   0.840
E M  3K2MD                 90    0.540 108.074  -1.023   0.004   0.197   0.799
E Y  3K2MD                 91    0.346  74.004  -1.572   0.005   0.336   0.660
E S  3K2MD                 92    0.413  48.439  -0.036   0.004   0.514   0.481
E P  3K2MD                 93    0.304  43.180  -0.590   0.003   0.718   0.279
B I  3K2MD                 94    0.087  16.040  -0.003   0.001   0.900   0.099
E S  3K2MD                 95    0.265  31.081   0.421   0.001   0.959   0.040
B I  3K2MD                 96    0.183  33.947   0.464   0.001   0.959   0.040
E N  3K2MD                 97    0.452  66.144   0.429   0.001   0.959   0.040
B Y  3K2MD                 98    0.146  31.179  -1.165   0.002   0.816   0.182
E R  3K2MD                 99    0.382  87.478   0.084   0.003   0.718   0.279
E T  3K2MD                100    0.357  49.474  -0.622   0.004   0.514   0.481
E C  3K2MD                101    0.536  75.311  -1.460   0.016   0.005   0.979

Explain the output.  Go back.
```

---
